# Interrater and Intrarater Reliability of the EasyForce Dynamometer for Assessment of Maximal Shoulder, Knee and Hip Strength

**DOI:** 10.3390/diagnostics12020442

**Published:** 2022-02-09

**Authors:** Nebojša Trajković, Žiga Kozinc, Darjan Smajla, Nejc Šarabon

**Affiliations:** 1 Faculty of Sport and Physical Education, University of Nis, 18000 Nis, Serbia; nele_trajce@yahoo.com; 2 Faculty of Health Sciences, University of Primorska, 6310 Izola, Slovenia; ziga.kozinc@fvz.upr.si (Ž.K.); darjan.smajla@fvz.upr.si (D.S.); 3 Andrej Marušić Institute, University of Primorska, 6000 Koper, Slovenia; 4 Human Health Department, InnoRenew CoE, 6310 Izola, Slovenia; 5 Laboratory for Motor Control and Motor Behavior, S2P, Science to Practice, Ltd., 1000 Ljubljana, Slovenia

**Keywords:** dynamometry, reliability isometric strength, upper limb, lower limb

## Abstract

This study aimed to determine the interrater and intrarater reliability of EasyForce dynamometer for assessing shoulder, knee, and hip muscle strength in healthy young adults. Shoulder, knee, and hip maximal isometric strength were measured using the EasyForce in healthy adults (11 women and 12 men). Three repetitions of shoulder internal rotation, abduction, knee flexion, extension, and hip abduction and adduction were performed. The tests were performed by three raters on the same day. The results showed good to high inter- and intrarater reliability (intraclass correlation coefficient range: 0.63–0.91). Moreover, the absolute reliability of the EasyForce was slightly higher than acceptable for all tests (CV > 10%) except for hip abduction on the right leg (CV = 7.2%). The EasyForce dynamometer can be considered a reliable tool for assessing shoulder internal rotation and abduction, knee extension and flexion, as well as hip abduction and adduction strength. The EasyForce dynamometer showed no differences between the raters’ measurements, which could be of great importance for professionals who want to perform the tests regardless of their strength on the values.

## 1. Introduction

The assessment of muscle strength has received a lot of research attention in areas where it is important to analyze the health and physical status of individuals [1]. In sports science, muscle strength assessment is primarily done for the purpose of setting normative standards for inclusion in certain sports [2,3], identifying and selecting potential talents [2], improving physical performance [4], or determining the effects of the training process [5]. Medical science requires strength assessment in the rehabilitation process following surgical interventions [6], to detect the risk of potential injury [7], assess the status of patients with neurological diseases [8,9] or disease’s progression [4,6], and set the normative standards of muscle strength levels for the general population [10]. For adequate and widespread strength assessment, it is of great importance to providing valid, reliable, discriminative, and practical equipment for that purpose [4,6,11,12].

The two most commonly used methods for assessing muscle system functionality are the manual muscle test (MMT) and an isokinetic dynamometer [4,6,12,13,14,15]. However, both of these approaches have weaknesses. MMT is successfully used in patients with neurological impairments and during the rehabilitation process [14]. Existing research has agreed that this method is inexpensive, fast, and easy to perform, but has failed to explore deficiencies in larger muscle groups [16] and minor strength deficits in relation to normal strength, since the ordinal rating scale of measurement is used in the assessment [17,18]. The ordinal rating scale is not applicable to all participants, especially to athletes who need accurate values on a test that MMT is unable to detect [18,19]. In contrast to MMT, strength testing using the isokinetic dynamometer is a more valid and reliable method [20,21]. However, this instrument is expensive and requires more space as well as professionally trained examiners [12,21,22,23]. Moreover, using the isokinetic dynamometer for testing multiple joints requires both time and a change of device configuration, making the testing impractical for the assessment of a large number of joints.

An alternative option for measuring muscle strength are hand-held dynamometers (HHD), which have been used extensively [24,25,26]. Previous studies [26,27] provided empirical evidence supporting HHD as a portable and less expensive alternative to the isokinetic dynamometer. Additionally, the measurements of isometric force with HHD have been shown to be safe for participants of different ages [16,28,29,30], activity levels [27,30,31], and health states [28,32]. There seem to be several limitations associated with HHD. One of the most important is the insufficient strength of the tester during the examination of larger muscle groups [21]. This leads to insufficient isolation of the tested muscle and inaccurate results [21]. A recent review [33] has argued that high levels of interrater reliability are only possible if the strength of the tester is superior to the strength of the subjects. Another problem that negatively affects the reliability is the diversity of devices and measurement protocols [11,28,29]. In addition, the population, age, and anthropometric characteristics of the respondents should be taken into account [27] because it is impossible to generalize or compare the results without considering these factors [16].

EasyForce is a new and effective tool for the measurement of muscle force production. EasyForce is purported to allow practitioners to measure muscle force objectively, and thus reducing the need for subjective measures and providing valuable data to aid in the design of treatment plans. Moreover, Easyforce is a dynamometer specifically designed for belt-stabilized HHD, which is of great importance, having in mind that stabilizing dynamometers with belts has shown better reliability and validity [34,35]. However, the vast majority of literature on HHD has focused on devices that are not integrated within the belt. We have recently assured the intervisit reliability of the EasyForce for assessing knee and hip muscle strength [36]. On the other hand, inter-rater reliability is often considered as the most problematic for HHD measurements. However, no study has looked specifically at interrater reliability of EasyForce HHD. To help fill this gap in the literature, this study aimed to examine the interrater and intrarater reliability of the EasyForce device to measure the strength of different muscle groups, using a standardized protocol among young adults. We hypothesized that the EasyForce will show good reliability for all assessments.

## 2. Materials and Methods

### 2.1. Participants

The study included 23 healthy young participants (12 males, 11 females; age: 21.4 ± 2.1 years) who reported being active in their leisure time. Mean body height was 1.82 ± 0.09 m for males, and 1.69 ± 0.06 m for females, and the mean body mass was 78.1 ± 8.9 kg for males, and 60.1 ± 6.2 kg for females. The inclusion criteria were the absence of injuries in the past 6 months and the absence of other medical conditions. All participants were thoroughly informed about the experimental procedures and signed an informed consent form before starting with the tests. The experimental protocol was approved by the Republic of Slovenia National Medical Ethics Committee (approval no. 0120-99/2018/5) and was performed in accordance with the latest revision of the Declaration of Helsinki.

### 2.2. Study Design and Procedures

**Figure 1:**
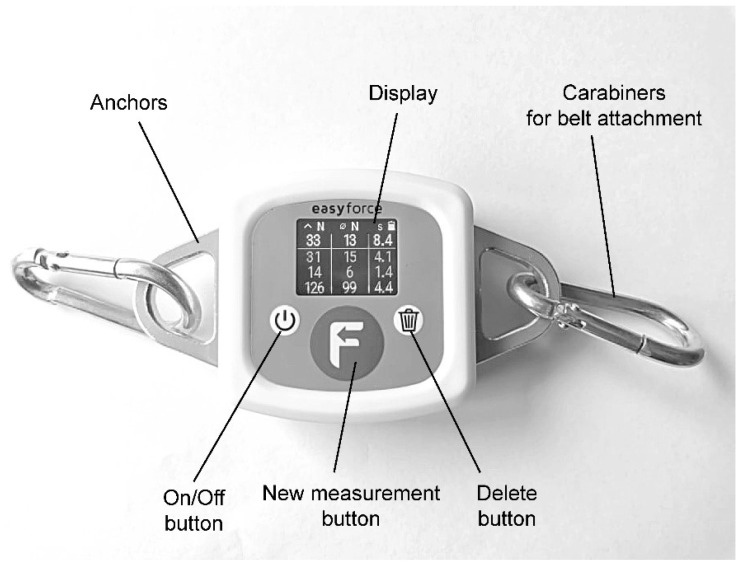
EasyForce dynamometer.

All measurements were performed by three raters with a background in kinesiology, who were familiarized with the procedures before the measurements. Rater 1 was male, height, 1.70 m; and weight, 68 kg; rater 2 was female, height, 1.67 m; weight, 59.5 kg; and rater 3 was female, height, 1.70 m; weight, 58.5 kg. The raters undertook a period of training and familiarization in the use of the EasyForce HHD (Figure 1) to ensure competency and efficiency. In addition, a pilot study was carried out on two participants prior to the commencement of testing. Throughout the testing period, each rater was blinded to the values obtained by the other raters. The EasyForce device continuously records the pulling force (with ±1% accuracy as per the manufacturer). After the force level is dropped below zero value, the data acquisition is stopped, and the results are displayed on the device. A new measurement is commenced after pressing the reset button. This is important, as it neglects any additional forces caused by movements after the force is dropped to zero.

Before performing the testing, the anthropometric characteristics of the participants were measured. Shoulder, hip, and knee strength measurements were taken with EasyForce dynamometer within the same visit. Prior to the measurements, participants performed a 15 min warm-up consisting of a stationary bike ride for five minutes, followed by 10 min of dynamic stretching exercises and bodyweight resistance exercises (lunges, squats, push-ups, glute bridges).

The order of the tasks was randomized across participants, but the order of the tasks was constant for each rater in assessing individual participants. The order of the raters was also randomized for each participant. All assessments were performed on both legs. For all tasks, three trials were performed on each side with 30 s rest in between. Prior to each task, the subjects performed three warm-up trials at submaximal intensity (~50, ~70, and ~90% of self-perceived maximal effort) to familiarize themselves with the task. During the measurements with the EasyForce, the instruction was to build up the maximal force gradually (~1–2 s) and sustain it for an additional ~3–4 s. Verbal encouragement was given throughout the tasks. After completion of the practice trials, subjects completed three trials on each leg/arm, and measures were recorded.

### 2.3. Set-Up for the EasyForce Measurement

**Figure 2:**
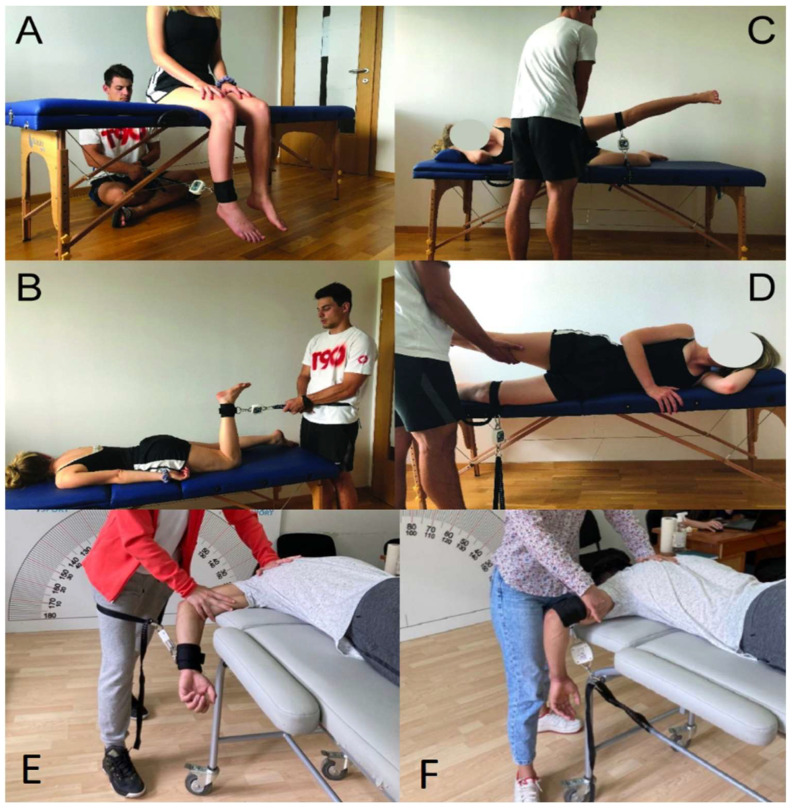
The positions for EasyForce measurements for knee extension (**A**), knee flexion (**B**), hip abduction (**C**), hip adduction (**D**), shoulder internal rotation (**E**), and shoulder abduction (**F**).

Measurements with the EasyForce dynamometer were performed according to the manufacturer’s recommendations (Figure 2). The EasyForce is a belt-stabilized HHD that continuously records tension force, with ±1% accuracy as assured by the manufacturer. After the force is dropped to zero, the measurement is terminated, and peak and average force are displayed. A new measurement is started only after resetting the device, which prevents any small movements performed after the measurement (i.e., after the force reaches zero) to influence the recorded peak and average values.

For the knee extension assessment (Figure 2A), the participants were seated on a bed table, with the knee flexed to 90°, hands resting on the thighs and the trunk in an upright position. The dynamometer was placed 2 cm above the malleolus, and the examiner was positioned behind the participant. For the assessment of knee flexion strength (Figure 2B), the participants were in a prone position on the table with the tested knee flexed to 90° and the dynamometer placed at the same point on the body as for the knee extension. While the dynamometer was fixed to the floor with the examiner’s foot, the hip abduction strength was assessed in the side-lying position (Figure 2C). The knee of the non-tested leg was flexed to 90°, while the upper leg was extended. The dynamometer was placed 2 cm above the lateral condyle, with the other end attached to the table. The examiner stabilized the pelvis during the measurements by holding it with both hands. For the assessment of hip adduction strength (Figure 2D), the same position was adopted, with the dynamometer fixed to the bottom leg with one end and the other end firmly attached to the bed table frame. Both legs were extended, and the examiner supported the upper leg, which was in slight abduction.

For the shoulder measurements (shoulder abduction and internal rotation), participants were in the prone position with their toes, abdomen, chest, and mentum touching on the portable table (Figure 2E,F). For the shoulder internal rotation (Shoulder IR) (Figure 2E), the placement of HHD positioned the transducer head just proximal to the ulnar styloid process on the ventral forearm. For shoulder abduction (Figure 2F), the person was prone with the shoulder abducted to 90° and elbow flexed to 90° with the upper arm resting on the table. The upper arm, shoulder, scapula, and trunk were stabilized by manual fixation by the examiner’s hand, arm, and trunk, if necessary.

### 2.4. Data Analysis and Statistics

Statistical analyses were done with SPSS (version 25.0, SPSS Inc., Chicago, IL, USA). Descriptive statistics are reported as mean ± standard deviation. Intra-class correlation coefficients (ICC) with the two-way random single-measure model (i.e., ICC_2,1_) for absolute agreement was used to assess the relative reliability of our outcomes. We considered ICC values < 0.5 as indicative of poor reliability, values between 0.5 and 0.75 for moderate reliability, values between 0.75 and 0.9 for good reliability, and values greater than 0.90 for excellent reliability [37]. Our previous study that assessed intervisit reliability of EasyForce for knee and hip muscles showed mostly excellent reliability (ICC > 0.90), therefore, we expected the ICC scores for this study to be >0.0. According to the recommendations by Bujang et al. [38], a sample of 18 participants would be needed to assure with 90% statistical power that the reliability is excellent (ICC > 0.90; the alternative hypothesis being that the reliability is below the good threshold; ICC < 0.75). Because interrater reliability could be lowest than intervisit reliability, we increased the sample size to 23. Absolute reliability was assessed with typical error [39], expressed as coefficient of variation (CV). Based on previous studies, the acceptable boundary of <10% for acceptable reliability was used for CV. Second, for the analysis of the agreement between the raters and to assess systematic between-rater bias, that is, if values obtained by one rater systematically differed from that of another rater, ANOVA was used. Values were expressed as mean ± SD and 95% confidence interval (CI). The significance level was set at α < 0.05.

## 3. Results

Table 1 shows the isometric strength data reported by the EasyForce HHD during the assessed movements of the shoulder, knee, and hip joint.

### 3.1. Interrater Reliability

**Figure 3:**
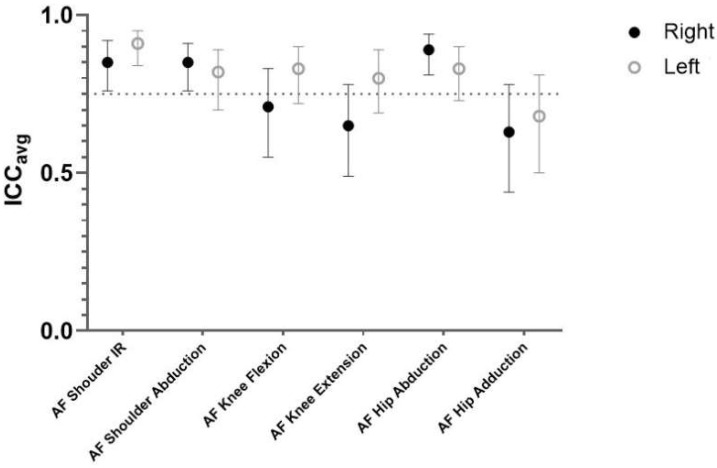
Relative interrater reliability of EasyForce for the right and left side.

Interrater reliability results are presented in Table 2 and Figure 3. Interrater ICC values ranged from 0.82 to 0.91 for shoulder, 0.65 to 0.83 for knee, and 0.63 to 0.89 for hip (Figure 3), showing moderate to excellent relative reliability. One-way ANOVA results also showed there were no significant differences between raters for all resistance muscle tests indicating no systematic bias. However, there was a high within-individual variation (CV = 11.24–23.54%) for all tests.

### 3.2. Intrarrater Reliability

**Figure 4:**
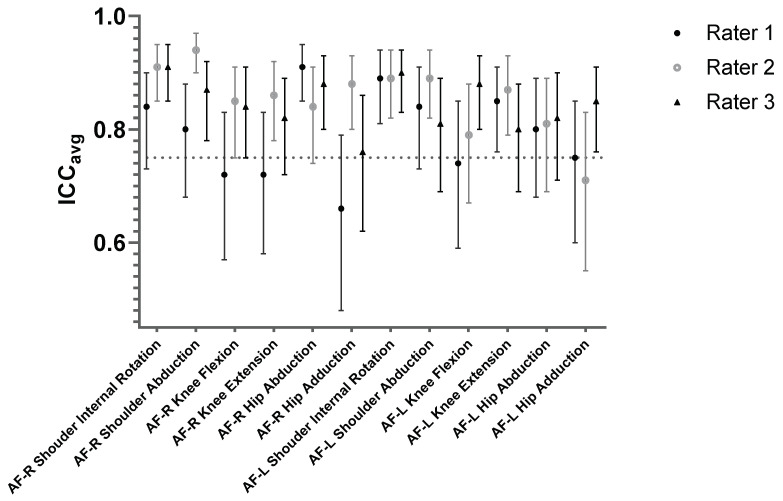
Relative intrarater reliability of EasyForce for three raters.

Intratester reliability results are presented in Table 3 and Figure 4. The ICC values obtained by rater 1 for all tests and both limbs ranged from moderate to excellent (0.66–0.91). Good to excellent reliability was also indicated by the highest ICC values obtained by rater 3 (0.76–0.91).

The absolute reliability (Table 3) of the EasyForce was slightly higher than acceptable (CV > 10%) with the exception of hip abduction on the right leg (CV = 7.2%). Moreover, one-way ANOVA results showed significant differences (*p* < 0.05) in several tests and both sides indicating systematic bias. For the knee measurements, there were significant differences in rater 1 (right knee flexion *p* = 0.046; eta squared (η^2^) = 0.103; left knee extension *p* = 0.021, η^2^ = 0.137), as well as for knee extension in both legs for rater 3 (right knee extension *p* = 0.001, η^2^ = 0.218; left knee extension *p* = 0.016, η^2^ = 0.126). Results for shoulder and hip showed significant differences (*p* < 0.05) in rater 1 left shoulder abduction *p* = 0.023, η^2^ = 0.136) and rater 2 (left shoulder IR *p* = 0.043, η^2^ = 0.121; left hip abduction *p* = 0.001, η^2^ = 0.242).

## 4. Discussion

The purpose of this study was to evaluate inter- and intrarater reliability of the new, portable, dynamometer EasyForce for assessing the isometric strength of shoulders, knee, and hip muscles. Overall, the results demonstrate moderate to excellent inter- and intrarater reliability with high within-individual variation for average peak torques in all tests.

Traditionally, shoulder internal rotation strength is assessed in a seated [40] supine [41] or prone position [40]. All shoulder joint measures of isometric strength used in this study demonstrated clinically acceptable levels of interrater reliability (ICC 0.82–0.91) using the prone position. Inter- and intrarater reliability results for shoulder isometric strength measures were similar to those of Hayes et al. and Cadogan et al. [42,43] for abduction (ICC = 0.84–0.96). In addition, isometric testing of the shoulder abductors using the HHD has shown excellent reliability in patients with shoulder pain (ICC = 0.77–0.98) [43] and in an asymptomatic university population (ICC = 0.94) [44]. Regarding shoulder IR, Katoh [45] found high ICC values (>0.9) in examining the test-retest reliability for HHD. Despite the high ICCs for shoulder measurements, our results revealed some systematic differences between trials and low absolute interrater reliability (CV > 10%).

Differences between trials for raters may be due to possible alterations in their technique following the performance of the initial trial. Moreover, reliable assessments with HHD require that the participants’ strength does not exceed the strength of the raters [46]. Despite satisfactory reliability (ICC > 0.80) for all measurements, current results revealed some systematic differences between trials as well. Nevertheless, it can be assumed that taking three measurements are sufficient to account for trial-to-trial variability. Therefore, the validity of the EasyForce is yet to be confirmed due to the abovementioned limitations and certain differences in testing positions.

Knee isometric strength tests have been widely used to estimate knee joint strength. Although the results of strength assessments are reported with different units of measurement, the data from this investigation can be compared to other studies. Previous studies conducted with or without a stabilization device reported moderate to high reliability for the assessment of isometric knee strength [26,47,48,49]. Studies that used HHD without a stabilization device reported lower inter- and intrarater reliability values [47,48]. This is in line with the current results regarding knee flexion and extension, where ICC values were from 0.65 to 0.83, showing moderate to good relative reliability. Additionally, absolute inter- and intrarater reliability were slightly over the acceptable threshold for knee measures (CV > 10%). Similarly, previous studies [26,50] also showed CV values higher than 10%. In a study by Lu et al. [50], the interrater CV ranged between 21.3 and 42.5% for the HHD without stabilization. On the contrary, Martins et al. [26], using the belt stabilization, showed slightly lower values for the absolute reliability for the knee strength assessment (CV = 12.0–22.0%). We can assume that better results for the CV and reliability could be achieved with more experienced raters. Therefore, it could be recommended that clinicians and practitioners practice the test extensively before applying it to patients or at least until they reach an acceptable CV, which is lower than 10%. Nevertheless, according to the high inter- and intrarater relative reliability results, we can state that EasyForce provides reliable data for assessing the isometric muscle strength of knee flexors and extensors, which supports the use of the dynamometer.

The interrater and intrarater reliability of HHD in assessing isometric hip strength has previously been established in healthy subjects [49,51,52]. In the study by Kollock et al. [51], intrarater reliability values ranged from 0.70 to 0.94 in the assessment of hip muscles. Maffiuletti and Mentiplay et al. [49,52] have also demonstrated good-to-excellent reliability for assessing the strength of the hip muscles. The current study also demonstrated moderate to high relative inter- and intrarater reliability for hip muscles ranging from 0.63 to 0.89. Studies that have investigated the reliability of HHD for measuring hip strength reported low reliability due to uncomfortable positions where stabilization of the pelvis is more difficult, like in prone and standing positions [47,53]. Accordingly, it was reported that the side-lying position (EasyForce measurements) provides the most valid hip abduction strength measurement [54], with slightly higher values obtained compared to the supine position.

This study had several strengths. The interrater reliability was carried out by three raters instead of two, which might have provided even more reliable information. We have also avoided the information bias since the raters were blinded from the strength values. The biggest limitation was that the sample consisted of a healthy population, limiting the generalization to other populations. Therefore, future studies should examine this protocol in clinical populations. Nevertheless, we consider the results of the current study important in providing normative values in healthy people.

## 5. Conclusions

The EasyForce dynamometer can be considered a reliable tool for assessing shoulder IR and abduction, knee extension and flexion, as well as hip abduction and adduction strength. Specifically, we found good to high intra- and interrater reliability with a slightly higher within-individual variation. The EasyForce dynamometer showed no differences between the raters’ measurements, which could be of great importance for professionals who want to perform the tests regardless of their strength on the values. Moreover, the biggest advantage of EasyForce is its affordability and portability, which allows the device to be used in different areas and settings.

## Figures and Tables

**Table 1:** Descriptive (mean ± SD) statistics for all the tests and raters.

Task		Rater 1			Rater 2			Rater 3		
		Repetition 1	Repetition 2	Repetition 3	Repetition 1	Repetition 2	Repetition 3	Repetition 1	Repetition 2	Repetition 3
Shoulder IR	right	95.9 ± 35.2	93.5 ± 33.3	100.0 ± 41.3	90.3 ± 39.2	86.0 ± 34.6	94.5 ± 41.7	91.6 ± 36.8	95.0 ± 34.3	93.0 ± 37.2
	left	91.2 ± 39.2	93.1 ± 41.0	88.4 ± 33.3	84.1 ± 33.7	93.1 ± 35.8	93.9 ± 43.0	87.5 ± 40.2	87.6 ± 38.8	89.6 ± 39.0
Shoulder Abduction	right	111.1 ± 53.4	111.2 ± 51.3	109.3 ± 52.9	100.0 ± 55.7	102.9 ± 60.5	109.1 ± 69.0	105.3 ± 47.4	101.0 ± 42.6	111.4 ± 45.2
	left	96.7 ± 42.6	106.4 ± 39.4	112.5 ± 46.9	104.0 ± 46.2	107.9 ± 50.6	108.0 ± 46.3	108.5 ± 45.0	109.5 ± 50.3	114.4 ± 50.8
Knee Flexion	right	93.5 ± 39.8	82.6 ± 23.1	84.4 ± 29.4	79.8 ± 44.8	80.4 ± 34.4	82.2 ± 31.1	81.1 ± 31.1	78.5 ± 30.5	80.4 ± 28.6
	left	78.4 ± 27.6	83.5 ± 29.5	82.5 ± 24.8	75.1 ± 24.8	80.5 ± 27.7	75.4 ± 24.7	77.2 ± 22.6	81.2 ± 24.0	80.2 ± 25.8
Knee Extension	right	193.0 ± 86.2	190.8 ± 69.8	199.9 ± 74.5	184.8 ± 60.4	204.5 ± 69.7	201.4 ± 69.2	186.3 ± 81.8	220.1 ± 81.1	223.4 ± 76.8
	left	161.4 ± 75.0	181.1 ± 70.8	187.7 ± 86.4	162.7 ± 65.1	178.9 ± 70.4	191.3 ± 63.7	176.8 ± 61.8	194.8 ± 79.2	199.1 ± 70.1
Hip Abduction	right	149.0 ± 45.4	146.7 ± 42.7	148.1 ± 48.6	137.3 ± 49.8	148.6 ± 56.9	148.3 ± 45.9	147.2 ± 48.5	151.7 ± 57.4	149.9 ± 55.9
	left	139.9 ± 49.9	135.5 ± 34.6	137.0 ± 39.2	120.9 ± 45.9	141.7 ± 40.1	145.2 ± 54.6	139.5 ± 56.0	141.3 ± 47.6	146.7 ± 57.3
Hip Adduction	right	81.5 ± 37.6	88.5 ± 35.0	94.0 ± 28.6	85.2 ± 45.1	99.5 ± 51.4	96.0 ± 43.6	81.4 ± 26.6	78.8 ± 25.7	83.4 ± 29.1
	left	77.0 ± 37.5	86.3 ± 38.5	83.5 ± 35.7	81.4 ± 35.8	78.5 ± 37.1	83.0 ± 44.9	73.1 ± 33.9	75.9 ± 32.3	83.4 ± 36.9

**Table 2:** Absolute interrater reliability for shoulder, knee, and hip isometric strength.

	Right							Left				
	TE	95% CI		CV	95% CI		TE	95% CI		CV	95% CI	
Shoulder IR	14.06	12.06	17.26	14.99	12.86	18.40	11.55	9.87	14.18	12.86	10.98	15.78
Shoulder Abduction	14.06	12.06	17.26	14.99	12.86	18.40	19.53	16.71	24.24	18.27	15.64	22.68
Knee Flexion	16.88	14.38	20.57	20.47	17.44	24.95	10.18	8.69	12.49	12.82	10.95	15.74
Knee Extension	39.95	34.53	48.82	20.07	17.35	24.52	27.93	23.89	34.33	15.76	13.48	19.37
Hip Abduction	16.57	14.22	20.35	11.24	9.65	13.80	18.79	16.05	23.06	13.55	11.57	16.63
Hip Adduction	19.51	16.64	24.13	22.72	19.38	28.10	18.68	15.93	23.45	23.54	20.07	29.55

Legend: shouder IR—shoulder internal rotation; CI—confidence interval; TE—typical error; CV—coefficient of variance (typical error expressed as a percentage of mean).

**Table 3:** Absolute intrarater reliability for shoulder, knee, and hip isometric strength.

		Right							Left				
	Rater	TE	95% CI		CV	95% CI		TE	95% CI		CV	95% CI	
Shoulder IR	1	15.39	13.20	18.89	15.94	13.68	19.58	13.18	11.24	16.30	14.49	12.36	17.92
	2	11.93	10.17	14.61	13.21	11.26	16.18	13.03	11.18	16.00	14.42	12.37	17.71
	3	11.15	9.50	13.59	11.95	10.18	14.57	13.09	11.23	16.07	14.82	12.72	18.20
Shoulder Abduction	1	24.10	20.68	29.60	21.80	18.71	26.77	18.03	15.37	22.30	17.12	14.60	21.18
	2	15.78	13.44	19.23	15.16	12.92	18.48	16.07	13.79	19.74	15.07	12.93	18.50
	3	17.15	14.65	21.25	16.18	13.83	20.05	22.06	18.79	26.98	19.91	16.96	24.27
Knee Flexion	1	17.13	14.70	21.04	19.71	16.92	24.21	14.26	12.16	17.63	17.49	14.92	21.63
	2	15.07	12.94	18.51	18.65	16.00	22.90	12.03	10.32	14.77	15.61	13.40	19.17
	3	12.29	10.47	14.98	15.35	13.08	18.71	8.63	7.41	10.60	10.85	9.31	13.33
Knee Extension	1	41.65	35.87	50.34	21.40	18.43	25.87	30.74	26.33	37.37	17.39	14.90	21.14
	2	25.45	21.77	31.15	12.92	11.05	15.82	22.48	21.05	29.99	13.78	11.85	16.88
	3	34.61	29.89	42.09	16.48	14.23	20.04	32.38	27.81	39.02	16.97	14.62	20.51
Hip Abduction	1	14.30	12.27	17.56	9.66	8.29	11.87	19.25	16.42	23.81	14.00	11.94	17.32
	2	21.20	18.19	26.03	14.64	12.56	17.98	21.41	18.38	26.29	15.74	13.51	19.33
	3	19.24	16.52	23.63	12.86	11.04	15.79	23.42	20.10	28.76	16.43	14.10	20.17
Hip Adduction	1	20.32	17.31	24.77	23.09	19.67	28.14	19.23	16.40	23.79	23.37	19.93	28.90
	2	16.91	14.51	20.77	18.07	15.51	22.19	21.81	18.72	26.78	26.91	23.10	33.05
	3	13.76	11.81	16.90	16.94	14.53	20.80	13.74	11.79	16.87	17.73	15.21	21.77

Shoulder IR, shoulder internal rotation; CI, confidence interval; TE, typical error; CV, coefficient of variance (typical error expressed as a percentage of mean).

## Data Availability

Not applicable.
